# The Interactive Effects between Particulate Matter and Heat Waves on Circulatory Mortality in Fuzhou, China

**DOI:** 10.3390/ijerph17165979

**Published:** 2020-08-18

**Authors:** Shumi Ji, Quan Zhou, Yu Jiang, Chenzhou He, Yu Chen, Chuancheng Wu, Baoying Liu

**Affiliations:** 1 Department of Preventive Medicine, School of Public Health, Fujian Medical University, Fuzhou 350108, China; jsm1020@126.com (S.J.); jiangyu@fjmu.edu.cn (Y.J.); hcz@fjmu.edu.cn (C.H.); zhwcy@fjmu.edu.cn (Y.C.); lby@mail.fjmu.edu.cn (B.L.); 2 Fuzhou Center for Disease Control and Prevention, Fuzhou 350000, China; 13358237946@163.com; 3 Fujian Provincial Key Laboratory of Environment Factors and Cancer, Fuzhou 350108, China

**Keywords:** heat waves, particulate matter, circulatory mortality, interactive effects

## Abstract

The interactive effects between particulate matter (PM) and heat waves on circulatory mortality are under-researched in the context of global climate change. We aimed to investigate the interaction between heat waves and PM on circulatory mortality in Fuzhou, a city characterized by a humid subtropical climate and low level of air pollution in China. We collected data on deaths, pollutants, and meteorology in Fuzhou between January 2016 and December 2019. Generalized additive models were used to examine the effect of PM on circulatory mortality during the heat waves, and to explore the interaction between different PM levels and heat waves on the circulatory mortality. During heat waves, circulatory mortality was estimated to increase by 8.21% (95% confidence intervals (CI): 0.32–16.72) and 3.84% (95% CI: 0.28–7.54) per 10 μg/m^3^ increase of PM_2.5_ and PM_10_, respectively, compared to non-heat waves. Compared with low-level PM_2.5_ concentration on non-heat waves layer, the high level of PM_2.5_ concentration on heat waves layer has a significant effect on the cardiovascular mortality, and the effect value was 48.35% (95% CI: 6.37–106.89). Overall, we found some evidence to suggest that heat waves can significantly enhance the impact of PM on circulatory mortality.

## 1. Introduction

Climate change and air pollution are the two major global public health challenges [1]. Every year, more than 3 million people die from air-pollution-induced disease worldwide, and most of these deaths are attributable to circulatory diseases [2,3]. Under the context of climate change, especially since the 2003 European heat wave event [4], numerous studies have found that high temperatures and heat waves were associated with an increased risk of mortality due to circulatory diseases [5,6,7,8,9]. Temperatures and particulate matter (PM) have independent effect on health, but the potential interaction due to PM and temperatures could also amplify the burden of disease [10]. However, to date, research relating to the potential interactive effects between heat waves and PM on circulatory mortality is limited.

It has been asserted that the joint effect of high temperatures and air pollution on health outcomes should be greater than its respective independent effect [8,9]. Current research has begun to pay more attention to the interaction between air pollution and high temperatures [11,12], however, inconsistent findings still exist. For example, Li G. et al. found that compared with moderate or low temperatures, high temperatures have a stronger correlation in Tianjin [13], but a study in Guangzhou showed that high temperatures and PM have no interactive effects on cardiovascular and cerebrovascular health [14]. The inconsistency may be associated with the differences in the level and composition of PM in different regions [15].

In China, explorations about the interactions between heat waves and PM on circulatory mortality often focused on cities such as Beijing, Hefei, and Tianjing, which are polluted heavily [10,13,16]. However, there is a paucity of studies exploring the interactive effects in slightly polluted cities with a humid subtropical climate in southern China. Evidence has shown that low-level PM exposure could cause an increased excess risk of circulatory mortality [17,18,19,20]. Therefore, analysis of the combined effects of heat waves and low-level PM exposure may further inform the development of intervention measures to reduce the burden of air pollution and heat-induced circulatory mortality.

Characterized by a humid subtropical climate, Fuzhou has been called an “oven” city because of its extremely hot summers, and is one of the hottest provincial capitals in China according to the National Climate Center of the China Meteorological Administration. Compared to some heavily polluted cities in northern China with a temperate climate, air pollution in Fuzhou is not a prominent problem. According to the Ministry of Ecological Environment of China, during 2015–2018 in Fuzhou, there were only 12 days with an Air Quality Index (AQI) exceeding the threshold of 100, which means that the air is polluted and may cause harm to human health [21]. AQI is a popular comprehensive indicator of overall air pollution level based on multiple air pollutants [22]. Given the improving air quality in China due to continuous interventions and the likely increasing extremely hot days in a warming climate, we selected Fuzhou as a case study to explore the interactive effect of PM and heat waves on circulatory mortality. Results of this study can provide references for future studies in other less polluted coastal cities with relatively mild subtropical humid climates.

## 2. Materials and Methods

### 2.1. Materials

To monitor the impact of air pollution on population health, a nationwide Air Pollution Impact Monitoring Project has been initiated by the National Institute of Environmental Health, Chinese Center for Disease Control and Prevention. Fuzhou was selected as one of the monitoring cities. In our research, the monitoring data on daily air pollution were based on a four-year data collection from 1st January 2016 to 31st December 2019 from seven air pollution monitoring stations in the Fuzhou Environmental Monitoring Center Station, and daily meteorological monitoring data were collected through daily monitoring by the Fuzhou Meteorological Bureau, which is part of the nationwide network of monitoring stations. They strictly implement the relevant national technical requirements. The indicators of air pollution included PM_10_ (particulate matter less than 10 μm in aerodynamic diameter) and PM_2.5_ (particulate matter less than 2.5 μm in aerodynamic diameter). The meteorological indicators included daily average air pressure, daily mean relative humidity, and daily maximum temperature. The daily data on mortality were obtained from the Fuzhou Center for Disease Control and Prevention (Fuzhou CDC), which has been part of the Fuzhou health monitoring network for four consecutive years, and was subject to strict quality control as per the requirements of the Air Pollution Impact Monitoring Project work manual. The causes of death were classified according to codes of the International Classification of Diseases, 10th edition (ICD-10), as follows: circulatory diseases (ICD10: I00-I99), cardiovascular diseases (ICD10: I05-09, I11, I20-I27, I30-52), and cerebrovascular diseases (ICD10: I60-I69). Among the I00-I99 codes, I05-09, I11, I20-I27, and I30-52 represent cardiovascular diseases and I60-I69 represent cerebrovascular diseases, which indicates that cardiovascular diseases and cerebrovascular diseases are part of the diseases of the circulatory system.

### 2.2. Statistical Analysis

Daily death, air pollution, and weather data are linked by date and therefore were analyzed with a time-series design [23]. Since daily mortality counts are a small probability event and follow a Poisson distribution, time-series Poisson generalized additive models (GAMs) were employed in the study [24]. Given that the value we aimed at obtaining was the risk of mortality on a heat wave day versus that same day without heat waves, we considered only those days that had a potential to have heat waves. Thus, we restricted our analysis to days in the summer season (May–October) from 2016 to 2019 [25]. Moreover, according to the China Meteorological Administration (2018), it is suggested by the World Meteorological Organization to define a heat wave when the maximum temperature exceeds 32 °C for three consecutive days [26,27]. First, in order to investigate the interaction between PM and heat waves, we used the GAMs to evaluate the risk of PM on the circulatory mortality under heat wave and non-heat wave layers. We adjusted the long-term trend of time, short-term fluctuations, and relative humidity. The natural spline (ns) function of date was also used in the GAMs to address nonlinear trends, sequence correlations, and the number of events per day on the time axis. The model is as follows:

logE (Yt) = β_K_Z_t_H_K_ + ns (time, df) + ns (Xt, df) + DOW + intercept

where E (Y_t_) is the expected value of the number of deaths due to circulatory diseases on day t; Z_t_ is the pollutant concentrations on day t; β is the exposure-response coefficient; H_K_ denotes the dummy variable for heat wave (H_1_ = 0, H_2_ = 0) and non-heat wave days (H_1_ = 0, H_2_ = 0); β_1_ and β_2_ represent the effect of PM in the heat wave layer and non-heat wave layer, respectively. ns () is the natural smoothing spline function; time is the calendar time variation; df represents the degrees of freedom; (the df) for date were 7 df per year [10]. X_t_ is the meteorological factor. Degrees of freedom of 3 were chosen for relative humidity [28]. DOW is the weekly variation; the day of the week (DOW) was considered in this model to control for the natural fluctuation trends over a particular week.

Second, for a better illustration of the effect modification, the effect of high and low levels of PM was also reported under heat wave and non-heat wave layers, respectively. High- and low-level PM days were defined as those where the daily average PM value was >50th and ≤50th percentile of the warm season from 2016 to 2019, respectively. (Note: 50th percentile of the distribution for PM_2.5_ was equal to 20 µg/m^3^, 50th percentile of the distribution for PM_10_ was equal to 52 µg/m^3^). The model is as follows:

logE (Yt) = β_K_Z_t_P_K_ + ns (time, df) + ns (Xt, df) + DOW + intercept

P_K_ denotes the dummy variable for high (P_1_ = 0, P_2_ = 0) and low (P_1_ = 0, P_2_ = 0), β_1_ and β_2_ represent the effect of PM in high and low PM levels, respectively. We examined a single-day lag (from lag0 to lag7) and the cumulative lag effect (from lag0–1 to lag0–7) in the GAMs. We tested for differences in effect estimates between different layers by calculating the 95% confidence intervals (CI) as shown below:

$$\left(Q_{1}-Q_{2}\right)\pm 1.96\sqrt{{\left({\mathrm{SE}}_{1}\right)}^{2}+{\left({\mathrm{SE}}_{2}\right)}^{2}}$$

where Q_1_ and Q_2_ are the estimates of the two levels, and SE_1_ and SE_2_ are their standard errors, respectively [29]. A *p*-value of <0.05 was considered statistically significant.

All data analyses were performed using “mgcv” package in statistical software R version 3.6.1. The results are presented as the percent increase (i.e., $\mathrm{ER}\left(\mathrm{excessive}\;\mathrm{risk}\right)=\left(e^{10\times \beta }-1\right)\times 100\%\;$, $\mathrm{ER}\left(95\%\;\mathrm{CI}\right)=\left(e^{\left(\beta \pm 1.96\mathrm{SE}\right)\times 10}-1\right)\times 100\%$) in daily mortality for each 10 μg/m^3^ increment in PM concentrations.

## 3. Results

During the warm season from 2016 to 2019, there were 31 heat wave events in Fuzhou, including 339 days with heat waves and 397 days without heat waves. The earliest period of heat wave event was from 15 to 18 May, and the latest period was from 26 to 28 October. The longest heat wave event was from 12 July to 29 August in 2019, lasting 48 days. In 2017 and 2018, the occurrence of heat wave events was nine at most.

Descriptive statistics of daily circulatory mortality, PM, and meteorological conditions are presented in Table 1. The mean average PM concentrations were 56.06 μg/m^3^ for PM_10_, 21.69 μg/m^3^ for PM_2.5_ during heat waves.

**Figure 1:**
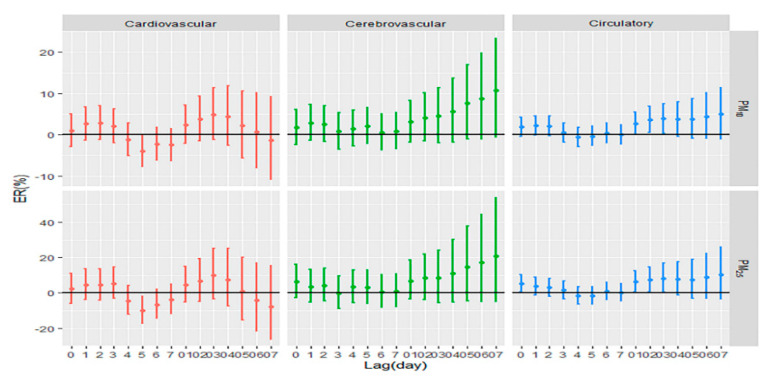
Estimates of percent change in daily circulatory mortality associated with a 10 μg/m^3^ increase in particulate matter concentrations during days with heat waves. Note: PM_10_: particulate matter less than 10 μm in aerodynamic diameter; PM_2.5_: particulate matter less than 2.5 μm in aerodynamic diameter; ER: excessive risk. X-axis: lag0 to lag7 means a single-day lag. lag0–1 to lag0–7 means cumulative lag effect.

Figure 1 shows the estimates of percent change in daily circulatory mortality (including cardiovascular mortality and cerebrovascular mortality) associated with a 10 μg/m^3^ increase in PM concentrations during days with heat waves. It was found that PM has a significant effect on circulatory mortality. We found that in lag0, lag0–1, lag0–2, and lag0–3, PM_2.5_ had a significant impact on the circulatory mortality. The effect values of lag0, lag0–1, lag0–2, and lag0–3 were 5.26% (95% CI: 0.35–10.40), 6.12% (95% CI: 0.32–12.27), 7.27% (95% CI: 0.50–14.49), and 8.21% (95% CI: 0.32–16.72), respectively. Simultaneously, we found that in lag0–1, lag0–2, and lag0–3, PM_10_ had a significant impact on circulatory mortality as well. The effect values of lag0–1, lag0–2, and lag0–3 were 2.73% (95% CI: 0.04–5.49), 3.63% (95% CI: 0.53–6.83), and 3.84% (95% CI: 0.28–7.54), respectively. From the results above, it can be concluded that PM has the maximum effect value on circulatory mortality at lag0–3. Therefore, the following analysis shows the effect values of lag0–3.

Table 2 shows that compared with the non-heat wave period, the effects of PM_10_ and PM_2.5_ on the circulatory mortality during the heat wave period are significantly increased. The effect values were 8.21% (95% CI: 0.32–16.72) and 3.84% (95% CI: 0.28–7.54), respectively.

Table 3 shows that compared with low-level PM_2.5_ layer on days without heat waves, the high level of PM_2.5_ concentration had a significant increase on the cardiovascular mortality. The effect value was 48.35% (95% CI: 6.37–106.89) for PM_2.5_.

## 4. Discussion

With the acceleration of urbanization, global-scale climate change will likely conspire in future years to increase population vulnerability to heat [30]. High temperature is related with global warming and urbanization, and will have a direct impact on the number of heat wave days in the future, increasing the potential for increased circulatory deaths in Fuzhou [19]. Given the improving air quality in China due to continuous interventions and the likely increase in extremely hot days in a warming climate, research in Fuzhou on the health hazards of high temperatures and PM may have important public health significance.

We used GAMs to examine the interactive effects of air pollutants and heat waves on circulatory mortality in Fuzhou, China. We estimated and compared the effect of heat waves on days with high- and low-level PM. We found some evidence that PM modifies the association between heat waves and circulatory mortality. Stronger heat wave effects were observed on days with high-level compared to low-level PM days for cardiovascular mortality. Our results are consistent with the positive PM–temperature interaction reported by Roberts [31], Ren et al. [32], Qian et al. [33], Stafoggia et al. [34], and Breitner [15], all concerning circulatory mortality in various parts of the world. This suggests that the human body is more susceptible to the toxic effects of PM during hot days [35]. In the present study, the effect value of the interaction between heat waves and PM on circulatory mortality is higher than that of the findings of Lin T. [16], in Beijing. Several previous studies have also found null or negative associations between increased temperatures or extreme heat and PM for circulatory mortality. A study in Guangzhou found that high temperatures and PM have no interactive effects on circulatory mortality [14]. A study in Shanghai found a statistically significant interaction between PM and lower temperatures in their effects on daily mortality, but no significant interaction between air pollution and higher temperatures was found [36]. The results above show that the effects of the interaction between PM and heat waves on death in different areas are different, which may be related to the level and source composition of PM differences across regions and cities [37,38], as well as to population acclimatization to temperature changes and heat waves [39,40]. In addition, the gender, age, and socioeconomic status of the population in different regions will play a role in the association between air pollution, temperature, and mortality outcomes. In the age group, some research found that the effect estimates of population mortality for older people are stronger than for the young under air pollution and high temperature conditions [41,42]. On a biological level, the physiological processes decline as people get older, especially the thermoregulatory ability [43]. A greater susceptibility has been reported for the people with underlying diseases and for those with a lower socioeconomic status [4,13], and could be related to poorer health status, limited access to health care, and poorer housing conditions in these socially disadvantaged groups [44]. In the gender group, some studies found the effect of heat mortality was stronger for females [45]. The gender-specific susceptibility might be due to biological (e.g., physiopathological responses to heat stress and air pollution) and behavioral difference (e.g., type of work—indoor/outdoor) between males and females [46,47]. It is therefore important to conduct further localized studies to account for these differences and clarify our understanding of any potential interactive effects of these environmental exposures on circulatory mortality.

We observed stronger heat wave effects on days with high-level PM_2.5_ compared to those with low-level PM_2.5_ for cardiovascular mortality. High-level PM_2.5_ enhances the heat wave effect. These results are similar to the findings of Analitis [48]. There are various plausible explanations for a synergistic association between heat waves and air pollutants. A proportion of particles (secondary particles) is generated by processes in the atmosphere in the presence of sunlight and primary emitted pollutants. On hot days, emissions of pollutants may be further increased by behavioral changes (e.g., inhabitants of cities may choose to use their possibly air-conditioned car more often). In addition, differences in pollution sources could lead to pollutants with different characteristics (e.g., more toxic) [48]. Exposure to extreme heat may also make humans more susceptible to air pollutants by causing physiologic stress or exposure to high PM concentrations may make them more sensitive to the effects of heat [49].

It is plausible that air pollution and heat exposure may interact on a biological level, although the exact causal pathways and mechanisms involved are not yet fully elucidated. In high-level particles, human blood viscosity, and some albumin increase, causing thrombosis. Epidemiological studies have also shown that PM causes vascular endothelial cell death through oxidative damage, increasing the incidence of cardiovascular diseases and mortality. The activation of the body’s thermoregulatory system and mechanisms during heat stress can facilitate and increase the absorption and entry of toxins and air pollutants into the body, as well as alter the body’s response to such substances [35]. It is biologically plausible that high temperatures could exacerbate the toxic effects of many environmental toxicants, such as PM [49]. The strength of the toxicity of a chemical or toxin on a biological system can be exacerbated by increased body temperature. Passive heat exposure can stress the cardiovascular system, where increased skin blood flow during thermoregulation results in increased cardiac output, which in turn is mediated by increases in heart rate [50].

This study has a potential strength. To the best of our knowledge, this is the first study that examines the potential interactive effects between heat waves and PM on circulatory mortality from a city characterized by a humid subtropical climate and low level of air pollution in coastal areas of China. We have shown that circulatory mortality may be susceptible to the potential interactive effects of heat waves and PM, especially in the high-level PM concentrations on days with heat waves.

This study has some potential limitations. First, the analysis was performed in a single city, therefore, our results may not be generalizable, given that PM levels and mixtures can vary geographically, as well as due to population acclimatization to heat waves. Second, the period of observation is limited to four years. The effect estimates would be imprecise in such a limited period. As the study period increases, the results obtained will be more reliable. Third, we used population measures of air pollutant concentrations and temperatures rather than the personal exposure levels, which will underestimate the effect value [51]. Fourth, we used a single model in the study. In future research, we can explore combining different models to improve the accuracy of the results [52]. Given the several limitations above, we have limited power to detect robust association. Further, heat wave forecasts or government-issued heat wave warnings may result in individuals exhibiting avoidance behaviors, especially for people with existing health conditions. This individual-level response is beyond the scope of this research.

## 5. Conclusions

In the present study, we found that heat waves can significantly enhance the impact of PM on circulatory mortality. Compared with low levels of PM_2.5_, heat waves and high levels of PM_2.5_ have a greater influence on cardiovascular mortality. These findings suggest that improving air quality and developing strategies and policies for controlling and preventing temperature-related diseases would benefit health of the general population.

## Figures and Tables

**Table 1:** Summary statistics of daily circulatory diseases deaths, particulate matter, and meteorological factors in Fuzhou during days with heat waves and days without heat waves during the warm season, 2016–2019.

	Heat Waves	Non-Heat Waves
Days	339	397
Deaths (number of cases)		
Circulatory diseases	10.26 ± 3.37	10.27 ± 3.47
Cardiovascular diseases	3.89 ± 2.24	3.66 ± 1.95
Cerebrovascular diseases	3.02 ± 1.78	3.22 ± 1.77
Pollutants (ug/m^3^)		
PM_10_	56.06 ± 19.23	51.72 ± 24.25
PM_2.5_	21.69 ± 8.94	20.86 ± 9.63
Meteorological factors		
Daily maximum temperature (°C)	35.11 ± 1.58	28.75 ± 3.25
Mean pressure (hPa)	1002.06 ± 4.35	1006.57 ± 6.02
Mean relative humidity (%)	69.75 ± 6.84	74.12 ± 12.05

Abbreviations: PM_10_: particulate matter less than 10 μm in aerodynamic diameter; PM_2.5_: particulate matter less than 2.5 μm in aerodynamic diameter.

**Table 2:** Estimates of percent change in daily circulatory mortality associated with a 10 μg/m^3^ increase in particulate matter concentrations during days with heat waves and days without heat waves. The effects are presented as excessive risk with their corresponding 95% confidence intervals.

	PM_2.5_		PM_10_	
	Heat Wave Days	Non-Heat Wave Days	Heat Wave Days	Non-Heat Wave Days
Circulatory	**8.21** *	4.24	**3.84** *	1.29
	**(0.32, 16.72)**	(−3.29, 12.37)	**(0.28, 7.54)**	(−1.98, 4.66)
Cardiovascular	9.89	1.36	4.90	1.11
	(−3.56, 25.22)	(−10.08, 14.26)	(−1.19, 11.37)	(−4.09, 6.60)
Cerebrovascular	8.29	2.19	4.49	1.63
	(−5.58, 24.20)	(−9.57, 15.48)	(−1.95, 11.35)	(−3.75, 7.31)

Abbreviations: PM_10_: particulate matter less than 10 μm in aerodynamic diameter; PM_2.5_: particulate matter less than 2.5 μm in aerodynamic diameter. Bolded figures are statistically significant (*p* < 0.05). Figures marked with asterisk * are statistically significantly higher (*p* < 0.05) than equivalent estimates in the non-heat wave layer. The results show lag0–3 for PM_2.5_ and PM_10_.

**Table 3:** The effect of days with heat waves and days without heat waves on circulatory mortality with high levels of particulate matter compared to days with low levels of particulate matter. Effects are presented as excessive risk with their corresponding 95% confidence intervals ^a^.

		Low PM_2.5_	High PM_2.5_	Low PM_10_	High PM_10_
**Heat wave days**	Total days	155	184	146	193
	Circulatory	16.68	13.09	3.74	0.96
		(−23.59, 78.16)	(−7.16, 37.76)	(−20.01, 34.52)	(−8.21, 11.04)
	Cardiovascular	−32.95	**48.35** *	−23.6	14.73
		(−68.97, 44.86)	**(6.37, 106.89** **)**	(−51.23, 19.68)	(−2.97, 35.66)
	Cerebrovascular	53.54	9.4	52.85	−5.83
		(−27.66, 225.87)	(−23.41, 56.25)	(−3.79, 142.85)	(−20.72, 11.85)
**Non-heat wave days**	Total days	207	190	221	176
	Circulatory	−14.55	3.59	−4.51	−3.31
		(−38.60, 18.91)	(−12.54, 22.69)	(−14.93, 7.19)	(−12.32, 6.63)
	Cardiovascular	−36.24	−12.84	−6.44	−0.68
		(−62.69, 8.97)	(−32.81, 13.08)	(−22.90, 13.55)	(−15.07, 16.14)
	Cerebrovascular	3.58	29.94	−6.25	12.79
		(−38.73, 75.11)	(−3.67, 75.28)	(−22.72, 13.73)	(−4.60, 33.35)

Abbreviations: PM_10_: particulate matter less than 10 μm in aerodynamic diameter; PM_2.5_: particulate matter less than 2.5 μm in aerodynamic diameter. Bolded figures are statistically significant (*p* < 0.05). ^a^ “Low PM” means the daily average PM value is below the 50th percentile of data; “High PM” means the daily average PM value is above the 50th percentile of data. The PM_2.5_ value of the 50th percentile of data is 20 ug/m^3^. The PM_10_ value of the 50th percentile of data is 52 ug/m^3^. Figures marked with asterisk * are statistically significantly higher (*p* < 0.05) than equivalent estimates in the low-level PM_2.5_ layer during days without heat waves. The results show lag0–3 for PM_2.5_ and PM_10_.
